# Supplementary material for: Light-evoked deformations in rod photoreceptors, pigment epithelium and subretinal space revealed by prolonged and multilayered optoretinography
Source: Nat Commun. 2024 Jun 19;15:5156. doi: 10.1038/s41467-024-49014-5 (PMC11186825; doi:10.1038/s41467-024-49014-5)
Supplement: Supplementary file 1 — Supplementary Information [file 41467_2024_49014_MOESM1_ESM.pdf]

Supplementary Information

Light-evoked deformations in rod photoreceptors, pigment epithelium and subretinal space revealed by prolonged and multilayered optoretinography

Bingyao Tan[1,2†], Huakun Li[3†], Yueming Zhuo[4,5], Le Han[1,2], Rajeshkumar Mupparapu[1,2], Davide Nanni[3], Veluchamy Amutha Barathi[1,6,7], Daniel Palanker[4,8*], Leopold Schmetterer[1,2,3,6,7,9,10,11,12*] and Tong Ling[1,2,3,13*]

[1] *Singapore Eye Research Institute, Singapore National Eye Centre, Singapore.*

[2] *SERI-NTU Advanced Ocular Engineering (STANCE) Program, Singapore.*

[3] *School of Chemistry, Chemical Engineering and Biotechnology, Nanyang Technological University, Singapore.*

[4] *Hansen Experimental Physics Laboratory, Stanford University, Stanford, CA 94305, USA.*

[5] *Department of Electrical Engineering, Stanford University, Stanford, CA 94305, USA.*

[6] *Department of Ophthalmology, Yong Loo Lin School of Medicine, National University of Singapore and National University Health System, Singapore.*

[7] *Ophthalmology & Visual Sciences Academic Clinical Program (Eye ACP), Duke-NUS Medical School, Singapore.*

[8] *Department of Ophthalmology, Stanford University, Stanford, CA 94305, USA.*

[9] *Department of Ophthalmology, Lee Kong Chian School of Medicine, Nanyang Technological University, Singapore.*

[10] *Department of Clinical Pharmacology, Medical University of Vienna, Austria.*

[11] *Center for Medical Physics and Biomedical Engineering, Medical University of Vienna, Austria.*

[12] *Institute of Molecular and Clinical Ophthalmology, Basel, Switzerland.*

[13] *School of Electrical and Electronic Engineering, Nanyang Technological University, Singapore.*

† These authors contributed equally to this work

* To whom correspondence may be addressed.

E-mail: palanker@stanford.edu, leopold.schmetterer@seri.com.sg, tong.ling@ntu.edu.sg

Supplementary Discussion 1 - Characterization of decorrelation noise associated with out-of-plane motion during a minute-long recording

Supplementary Figure 1 - Representative three-dimensional eye movement measured in a minute-long recording.

Supplementary Figure 2 - The probability density function of the phase uncertainty resulting from out-of-plane motion (δy)

Supplementary Discussion 2 - Estimation of the hydraulic conductivity and water permeability coefficient of the rod OS membrane

Supplementary Table 1 - Parameters used in the estimation of the hydraulic conductivity

Supplementary Method 1 - Extraction of phase responses from the outer retina

Supplementary Method 2 - Calculation of rhodopsin bleaching

Supplementary Figure 3 - Flow chart for classifying phase traces in the mixed layer

Supplementary Figure 4 - Processing of phase traces and extraction of temporal signatures using principal component analysis

Supplementary Figure 5 - The distributions of three signal types in the spatiotemporal features space

Supplementary Figure 6 - Waveform of LED's response to trigger

Supplementary Table 2 - Animal Usage

Supplementary Table 3 - Acquisition protocols

Supplementary Discussion 1: Characterization of decorrelation noise associated with out-of-plane motion during a minute-long recording

To validate how phase stability can be maintained in the prolonged ORG protocol, we conducted an experiment to measure the three-dimensional eye movements over a minute-long recording. A cross-scanning pattern consisting of a horizontal scan (following the lateral direction in the repeated B-scans mode) and a vertical scan (following the out-of-plane direction in the repeated B-scans mode) was repeated over one minute. The temporal resolution was 45 ms, and the scanning areas in both directions were 12°. As demonstrated in Supplementary Figs. 1a-b, the eye movement in anesthetized rodents typically exhibited a superposition of a slow drift (<0.5 Hz) and periodic oscillations with multiple frequencies corresponding to breathing (~1 Hz) and heartbeat (~3 Hz). We separated the periodic oscillations (Supplementary Fig. 1c) and slow drift (Supplementary Fig. 1d) components by extracting their respective frequency bands (Supplementary Fig. 1b). We also calculated the residual displacements by subtracting both periodic oscillations and slow drift components from the measured displacements (Supplementary Fig. 1e).

We found that with a proper stereotaxic fixation of anesthetized rats, the peak-to-peak amplitude of the out-of-plane periodic oscillations could be reduced to within ±2 µm. Although we could not correct this out-of-plane movement through image registration, our previous study demonstrated that the decorrelation noise originating from a moving speckle pattern is a deterministic error rather than stochastic noise[1]. This suggests that the decorrelation noise, introduced by periodic oscillations at each pixel, exhibits a repetitive temporal pattern correlated with the oscillations, which can be filtered out if these oscillations stay within fixed frequency bands. Therefore, we employed bandstop filters corresponding to the frequencies and harmonics of the breathing effect and heartbeat in the subsequent data processing, which mitigated the impact of decorrelation noise caused by the periodic oscillations in the out-of-plane direction (see Supplementary Fig. 3b).

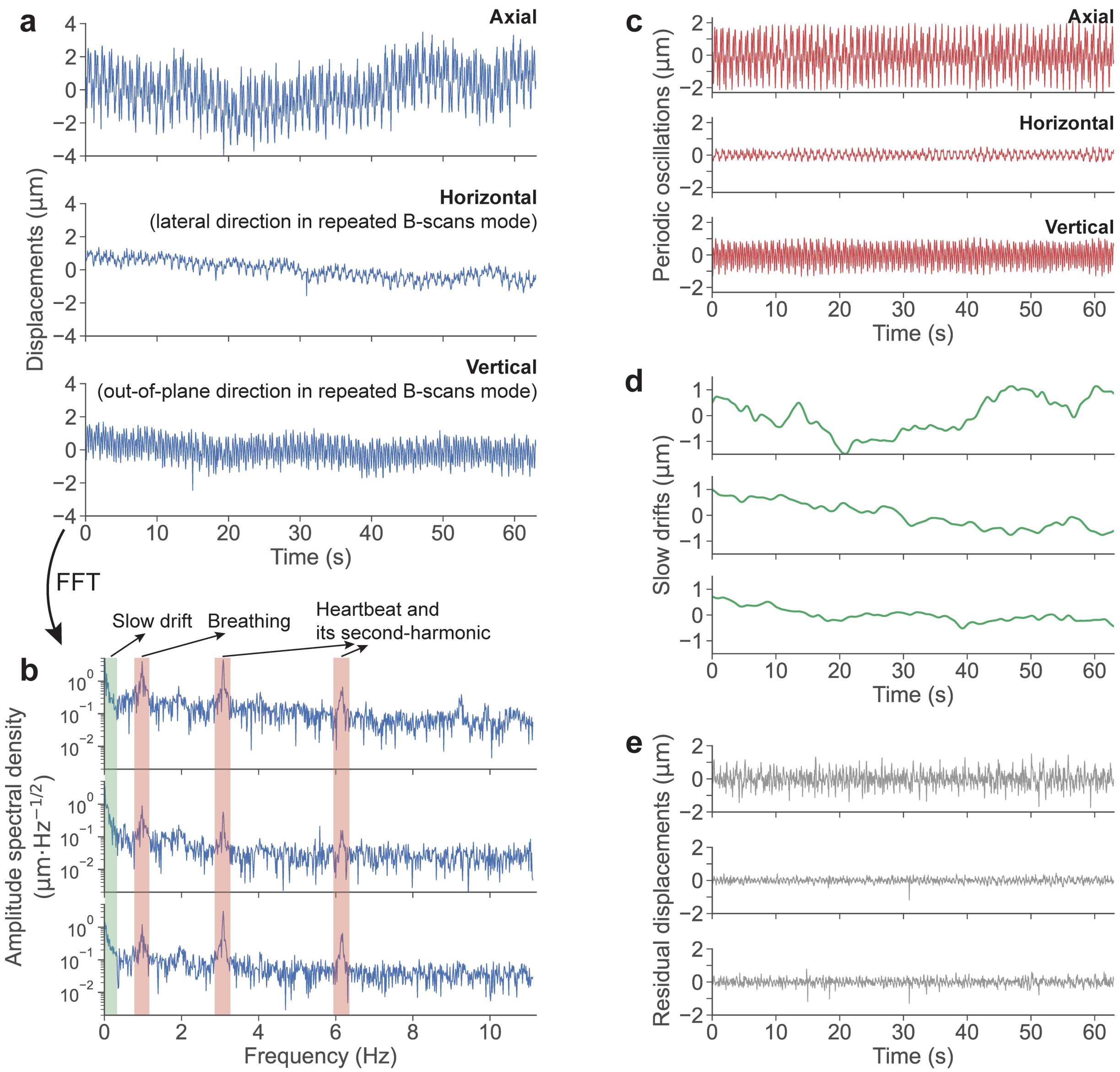

Supplementary Figure 1. Representative three-dimensional eye movement measured in a minute-long recording. **(a)** The displacements along the axial, horizontal (lateral direction in the repeated B-scans mode), and vertical (out-of-plane direction in the repeated B-scans mode) directions measured using the single-step DFT algorithm[2]. **(b)** The amplitude spectral densities of the displacements in (a) show distinct peaks representing periodic oscillations (indicated by red bars) and slow drifts (indicated by the green bar). The periodic oscillations corresponded to breathing (~1 Hz) and heartbeat (~3 Hz). **(c)** and **(d)** are the individual periodic oscillation and slow drift components, respectively, extracted from the corresponding frequency bands in (b). **(e)** Residual displacements obtained by subtracting both periodic oscillations and drifts from the measured displacements. Source data are provided as a Source Data file.

On the other hand, the out-of-plane slow drift may introduce non-oscillating phase uncertainty that is more challenging to correct. Nevertheless, in our experiments, such a drift could also be maintained within 2 μm over a 1-minute recording, which was much smaller than our OCT beam size (12.2 μm, theoretical $1/e^2$ diameter, potentially even larger due to ocular aberrations). To understand the scale of decorrelation noise caused by such out-of-plane drift, we further estimated the probability density function of phase uncertainty for different out-of-plane displacements using the model proposed in OCT velocimetry[3, 4]. As shown in Supplementary Fig. 2, a lateral drift of 2 μm would result in a 53% probability of phase uncertainty being less than 0.3 rad (~20 nm in optical path length) on a single pixel, which explains the marginal impact of out-of-plane drift on our measurements. Moreover, our proposed unsupervised learning approach enables averaging the signals across multiple pixels of the same signal type to further reduce phase uncertainty during the prolonged ORG recordings.

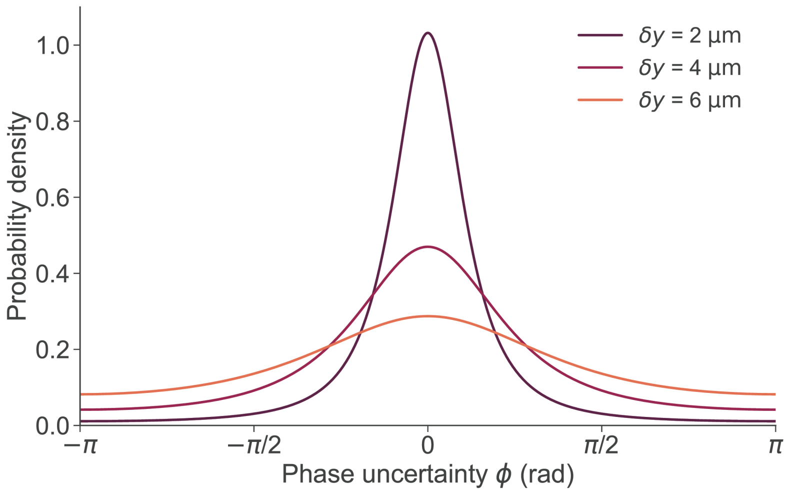

Supplementary Figure 2. The probability density function of the phase uncertainty resulting from out-of-plane motion (δy)[3, 4]. The theoretical diffraction-limited beam diameter at the $1/e^2$ level of the central maximum intensity was calculated to be 12.2 μm in our OCT imaging system. Source data are provided as a Source Data file.

Supplementary Discussion 2: Estimation of the hydraulic conductivity and water permeability coefficient of the rod OS membrane

We estimated the hydraulic conductivity and water permeability coefficient of the rod OS membrane based on a model developed by Zhang *et al.*[5]. Some parameters used in this model are summarized in Supplementary Table 1.

Supplementary Table 1. Parameters used in the estimation of the hydraulic conductivity

Parameters	Value	Unit
Normal rodent plasma osmolarity[6], Π_{rest}/RT	325	mOsM
Maximum elongation*, ΔL	33.6	nm
Length of the rod OS, L	24.0	μm
Surface area of the rod OS, S_{ROS}	132	μm^2
Cross-sectional area of the rod OS[5], A_{ROS}	1.54	μm^2
Initial water influx rate*, J_w	0.13	$\mu m^3 \cdot s^{-1}$

*: calculated with a refractive index of 1.41.

In brief, the model is based on osmotic swelling of the rod OS, which occurs in response to the incremental osmotic pressure triggered by the phototransduction cascade. Assuming that the saturated elongation is purely an osmotic equilibrium, the Van't Hoff's law indicates that the volume increase is proportional to the incremental osmotic pressure,

$$\Delta V_{\text{cyto}}/V_{\text{cyto, rest}} = \Delta\Pi/\Pi_{\text{rest}}, \quad \text{(S1)}$$

where Π_{rest} and $V_{\text{cyto, rest}}$ are the osmotic pressure and the cytoplasmic volume of the rod OS in the resting (dark-adapted) state. $\Delta\Pi$ is the phototransduction-induced increase in osmotic pressure, ΔV_{cyto} is the incremental cytoplasmic volume when a new osmotic equilibrium is established. Since a moderate osmotic perturbation does not alter the rod OS width and the disc membranes occupy ~50% of the rod OS volume[5], the fractional volume increase $\Delta V_{\text{cyto}}/V_{\text{cyto, rest}}$ can be estimated by $2\Delta L/L$. As a result, our measurements in Fig. 4b indicated that a flash with a bleach level of 0.28% increased the rod OS cytoplasmic osmolarity by $\Delta\Pi/RT = (2\,\Delta L/L)\cdot(\Pi_{\text{rest}}/RT)$ = 0.91 mOsM. Note that this value should be considered as a lower limit, because the restoring elastic force from expansion is ignored.

Considering the start of the phototransduction cascade, the water inflow rate is determined by the initial increase of osmotic pressure and the hydraulic conductivity L_p of rod OS membrane,

$$J_w = L_p S_{\mathrm{ROS}} \Delta\Pi, \tag{S2}$$

The initial water influx rate J_w can be calculated from the slope of the rod OS elongation. From our measurements, a 1-ms flash with a bleach level of 0.28% yielded an initial water influx rate of $8.5 \times 10^{-2}\ \mu\mathrm{m} \cdot \mathrm{s}^{-1}$ in length and $0.13\ \mu\mathrm{m}^3 \cdot \mathrm{s}^{-1}$ in volume (J_w) with the cross-sectional area of $1.54\ \mu\mathrm{m}^2$. Our measurements yielded a hydraulic conductivity of $4.2\ \mu\mathrm{m}^3 \cdot \mathrm{s}^{-1}$ and accordingly, a water permeability coefficient of $5.9 \times 10^{-3}\ \mathrm{cm} \cdot \mathrm{s}^{-1}$, which agrees with the in-vitro experiment results of $2.6 \times 10^{-3}\ \mathrm{cm} \cdot \mathrm{s}^{-1}$ measured by Preston *et al.*[7] and $> 2.0 \times 10^{-3}\ \mathrm{cm} \cdot \mathrm{s}^{-1}$ measured by Korenbrot *et al.*[8]. It should be noted that because the effect of restoring elastic force was ignored in the above model, the estimated hydraulic conductivity and water permeability coefficient should be considered the upper limits.

Supplementary Method 1: Extraction of phase responses from the outer retina

In phase-sensitive ORG measurements, we calculated the temporal phase change of a target layer with respect to a reference layer. For each pixel in the target layer, we selected a reference region from the reference layer centered at the same A-line. Each reference region comprised 5 adjacent A-lines (~3.9 μm laterally). Before conducting spatial averaging across the reference region, we canceled out the systematic phase drift by self-referencing and removed arbitrary phase offset of the individual phase trace by referring to its pre-stimulus frames. Specifically, the systematic phase drift was canceled out by calculating the multiplication of the complex-valued OCT signal of the pixel of interest in the target layer and the complex conjugate of the signals in the selected reference region:

$$\tilde{I}_{tar/ref}(s,i) = \tilde{I}_{tar}(i)\tilde{I}^{*}_{ref}(s,i), \tag{S3}$$

where $\tilde{I}_{tar}(i)$ is the complex-valued OCT signal of the pixel of interest in the target layer, i is the index of the frame number. $\tilde{I}_{ref}(s,i)$ represents signals in the corresponding reference region, and s denotes the pixel index in the reference region. $\tilde{I}_{tar/ref}(s,i)$ is the pairwise self-referenced signal, where the pixel of interest in the target layer was ergodically referred to all pixels in its reference region. $*$ represents complex conjugate.

To cancel out arbitrary phase offsets, each complex-valued signal trace was referred to its pre-stimulus frames,

$$\Delta\tilde{I}_{tar/ref}(s,i) = \tilde{I}_{tar/ref}(s,i) \cdot \frac{1}{N}\sum_{i=1}^{N} \tilde{I}^{*}_{tar/ref}(s,i), \tag{S4}$$

where $\Delta\tilde{I}_{tar/ref}(s,i)$ denotes time referenced signals, N represents the number of frames acquired before the light stimulus. The complex-valued signals were subsequently averaged across pixels in the reference region, and the phase information was extracted from the averaged complex-valued signal:

$$\Delta\tilde{I}_{tar/ref}(i) = \frac{1}{M}\sum_{s=1}^{M} \Delta\tilde{I}_{tar/ref}(s,i), \tag{S5}$$

$$\Delta\phi(i) = \angle\Delta\tilde{I}_{tar/ref}(i), \tag{S6}$$

where $\Delta\phi(i)$ is one phase trace extracted from the pixel of interest in the target layer. M is the total pixel number in the reference region, $\angle$ represents the calculation of argument.

We applied the same process to every pixel in the target layer to extract spatially resolved temporal phase traces. Note that when averaging phase traces across different pixels, calculations were performed in the complex plane to avoid biases introduced by phase wrapping[9].

Phase traces can be converted into the OPL change ΔOPL by:

$$\Delta \text{OPL} = \frac{\lambda_0}{4\pi} \Delta\phi, \tag{S7}$$

or

$$\Delta \text{OPL} = -\frac{\lambda_0}{4\pi} \Delta\phi, \tag{S8}$$

where λ_0 is the central wavelength of the OCT system. When the reference layer was anterior to the target layer, the OPL change was calculated using Eq. (S7). Otherwise, it was calculated using Eq. (S8). This ensured that an increase/decrease in OPL change (ΔOPL) consistently represented an expansion/contraction between the target layer and the reference layer.

Supplementary Method 2: Calculation of rhodopsin bleaching

Given the negligible regeneration of rhodopsin during a short light stimulus, rhodopsin bleaching can be calculated as following[10, 11]:

$$p(Q) = \exp(-Q/Q_e), \tag{S9}$$

where p is the fraction of unbleached rhodopsin present after the light stimulus, Q is the energy density of the 500 nm stimulus (photons/μm^2), Q_e is the energy density that reduces the fraction rhodopsin present to 1/e of its dark-adapted level, which was measured to be 7.94×10^7 photons/μm^2 in rats[10]. The energy density Q can be calculated as,

$$Q = \frac{Pt}{AE_v}, \tag{S10}$$

where P and t are the power and duration of the light stimulus, A is the illuminated area on the retina. E_v is the energy of a 500 nm photon and can be calculated by hc/λ, where h is the Planck constant, c is the speed of light in vacuum, λ = 500 nm is the photon's wavelength. Note that if the stimulus is broadband rather than just at 500 nm, additional calibration accounting for rhodopsin spectral sensitivity is required[11].

Supplementary Figures

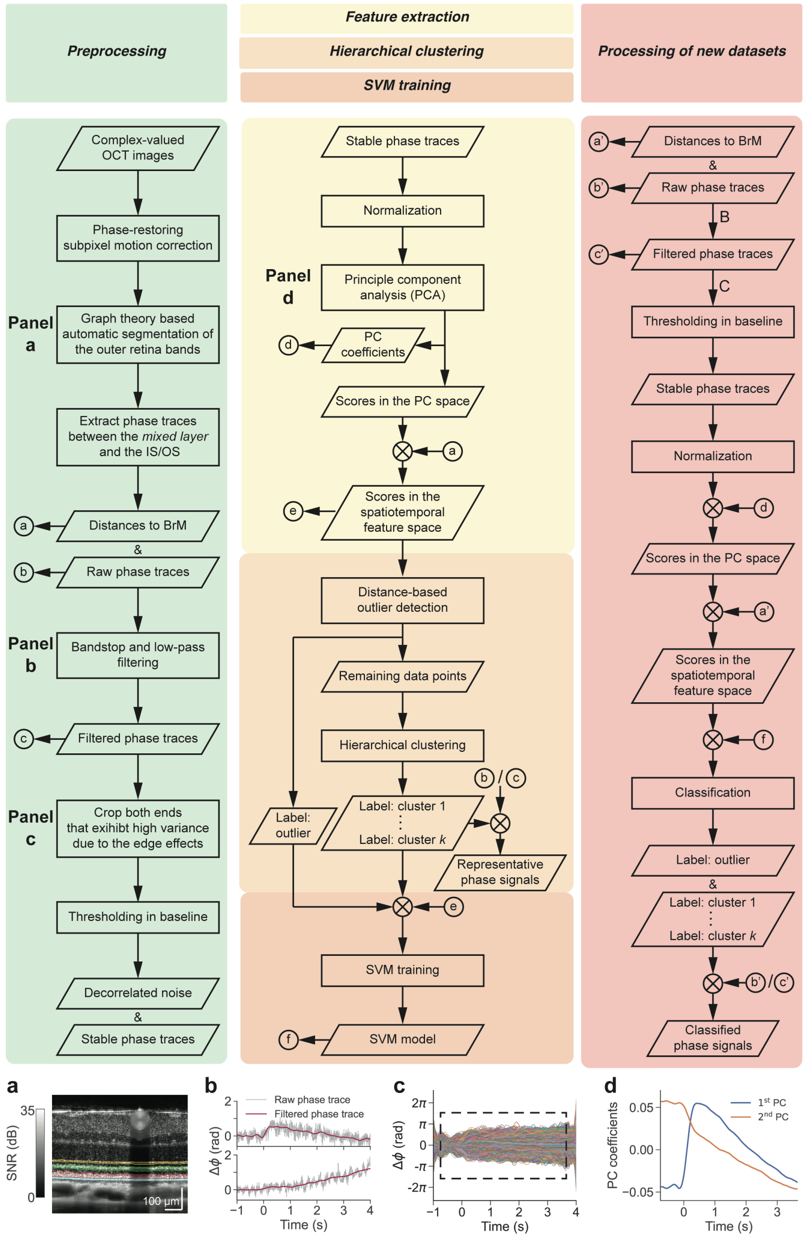

Supplementary Figure 3. Flow chart illustrating the classification of phase traces in the mixed layer. Phase traces extracted from the mixed layer were preprocessed and projected onto the spatiotemporal feature space. Distinct phase responses were identified using hierarchical clustering. A support vector machine was subsequently trained in the same feature space to facilitate the classification of new phase traces using the same criterion. **(a)** Hyperreflective bands segmented from the outer retina. The four colored bands from top to bottom were external limiting membrane (ELM), inner segment/outer segment junction (IS/OS), the mixed layer consisting of outer segment (OS) and retinal pigment epithelium (RPE), and Bruch's membrane (BrM). **(b)** Individual phase traces before and after filtering. **(c)** Both ends of filtered phase traces exhibiting high variance were removed from subsequent data analysis. **(d)** Coefficients of the top two principal components. Source data of panels b and d are provided as a Source Data file.

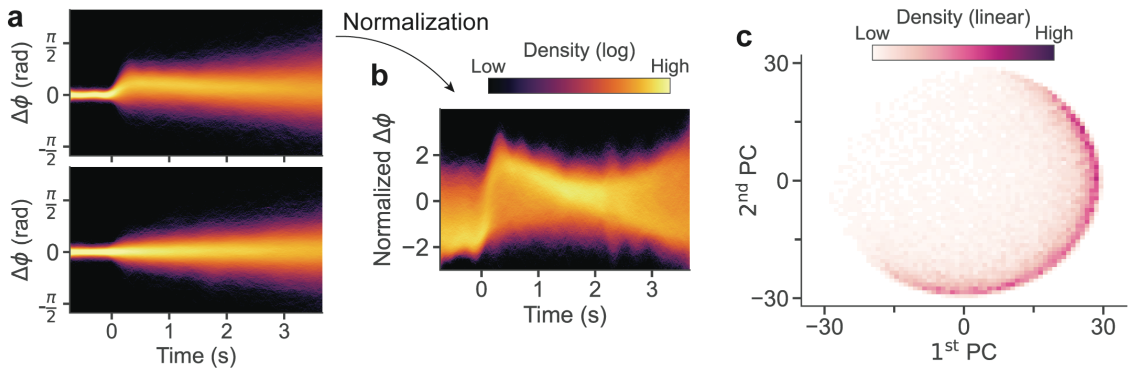

Supplementary Figure 4. Processing of phase traces and extraction of temporal signatures using principal component analysis. **(a)** The distribution density of phase traces that exhibited low variance prior to the light stimulus (before 0 s). When a flash was delivered to the retina (t = 0 s), function-associated phase responses were clearly observed (top panel), whereas without a light stimulus, phase traces gradually decorrelated without any noticeable response pattern (bottom panel). **(b)** Each phase trace in the top panel of Fig. S4a was then normalized by subtracting its mean value and divided by its standard deviation (SD). **(c)** The distribution of the normalized phase responses in the principal component (PC) space.

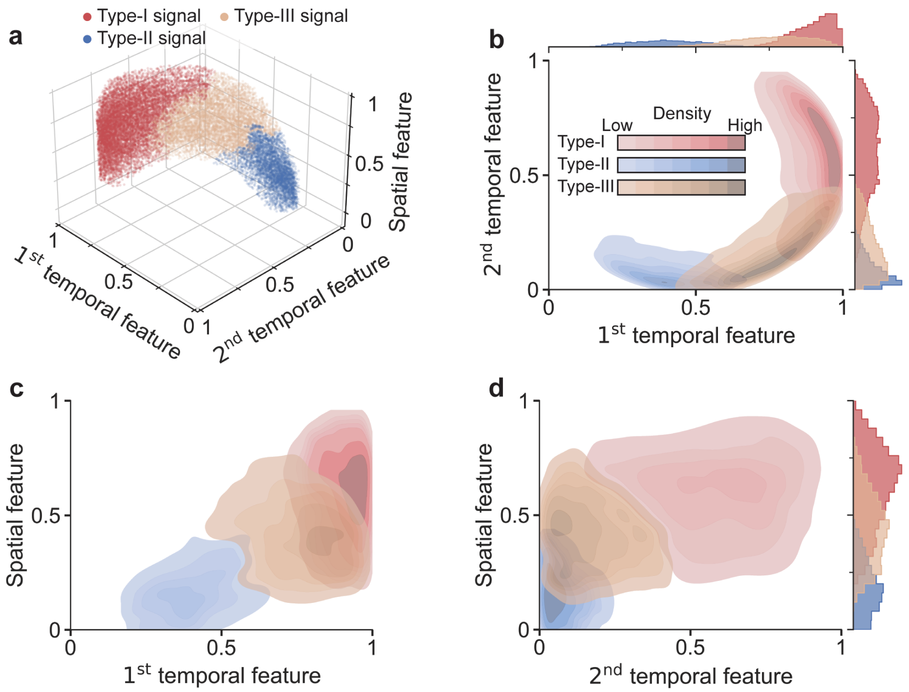

Supplementary Figure 5. The distributions of three signal types in the spatiotemporal features space. **(a)** The distributions of the Type-I (red), Type-II (blue), and Type-III (brown) signals in the spatiotemporal feature space, and **(b)-(d)** their projections onto 2D feature planes. The 1D histograms in (b) and (d) show the distributions of three signal types along each feature.

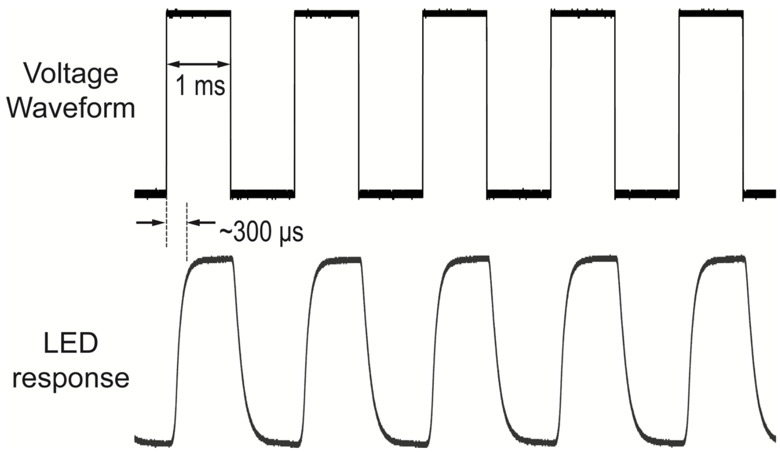

Supplementary Figure 6. Waveform of LED's response to trigger. Stimulus timing was delayed by approximately 300 μs, i.e., 6% of the B-scan frame acquisition time (5 ms). Source data are provided as a Source Data file.

Supplementary Table 2: Animal usage in ORG experiments

Test No.	Test Name	Number (Male)	Age
1	Pre-trained classifier	5 (3)	12 weeks
2	Dark adaptation	12 (10)	7-16 weeks
3	Light adaptation	15 (10)	6-12 weeks
4	Prolonged recording	9 (6)	6-12 weeks
5	Three-dimensional visualization	7 (4)	6-10 weeks

Supplementary Table 3: Acquisition protocols

**Test Number (refer to Table S2)**	**1**	**2**	**3**	**4**	**5**
Acquisition Mode	Repeated B-scan	Repeated B-scan	Repeated B-scan	Repeated B-scan	Repeated volumetric scan
Total Number of B-scans	1000	1000	1000	1375	1000
Number of B-scans per Volume	N/A	N/A	N/A	N/A	25
Total time of acquisition (s)	5	5	5	55	5
Time interval between acquisitions (min)	2	2	N/A	2	2
Light/Dark adaptation	Dark adaptation	Dark adaptation	Light adaptation	Dark adaptation	Dark adaptation
Background intensity (photons/(μm^2·s))	N/A	N/A	6×10^1 - 6×10^4	N/A	N/A
Flash duration (ms)	1	1	1	1	1
Flash bleach level (%)	0.10	0.0019 – 0.28	0.28	0.18 & 0.26	0.10

Supplementary References

1. Li H*, et al.* Shot-noise limited phase-sensitive imaging of moving samples by phase-restoring subpixel motion correction in Fourier-domain optical coherence tomography. *bioRxiv*, (2022).

2. Guizar-Sicairos M, Thurman ST, Fienup JR. Efficient subpixel image registration algorithms. *Opt Lett* **33**, 156-158 (2008).

3. Vakoc BJ, Tearney GJ, Bouma BE. Statistical properties of phase-decorrelation in phase-resolved Doppler optical coherence tomography. *IEEE Trans Med Imaging* **28**, 814-821 (2009).

4. Grafe MGO, Nadiarnykh O, De Boer JF. Optical coherence tomography velocimetry based on decorrelation estimation of phasor pair ratios (DEPPAIR). *Biomed Opt Express* **10**, 5470-5485 (2019).

5. Zhang P*, et al.* In vivo optophysiology reveals that G-protein activation triggers osmotic swelling and increased light scattering of rod photoreceptors. *Proceedings of the National Academy of Sciences*, 201620572 (2017).

6. Russell ES, Bernstein SE. Blood and blood formation. *Biology of the laboratory mouse* **2**, 351-372 (1966).

7. Preston GM, Carroll TP, Guggino WB, Agre P. Appearance of water channels in Xenopus oocytes expressing red cell CHIP28 protein. *Science* **256**, 385-387 (1992).

8. Korenbrot JI, Brown DT, Cone RA. Membrane characteristics and osmotic behavior of isolated rod outer segments. *The Journal of Cell Biology* **56**, 389-398 (1973).

9. Szkulmowska A, Szkulmowski M, Kowalczyk A, Wojtkowski M. Phase-resolved Doppler optical coherence tomography—limitations and improvements. *Opt Lett* **33**, 1425-1427 (2008).

10. Perlman I. Kinetics of bleaching and regeneration of rhodopsin in abnormal (RCS) and normal albino rats in vivo. *The Journal of Physiology* **278**, 141-159 (1978).

11. Zhang P, Goswami M, Zawadzki RJ, Pugh EN, Jr. The Photosensitivity of Rhodopsin Bleaching and Light-Induced Increases of Fundus Reflectance in Mice Measured In Vivo With Scanning Laser Ophthalmoscopy. *Investigative Ophthalmology & Visual Science* **57**, 3650-3664 (2016).
