# Supplementary material for: Light-evoked deformations in rod photoreceptors, pigment epithelium and subretinal space revealed by prolonged and multilayered optoretinography
Source: Nat Commun. 2024 Jun 19;15:5156. doi: 10.1038/s41467-024-49014-5 (PMC11186825; doi:10.1038/s41467-024-49014-5)
Supplement: Supplementary file 2 — Reporting Summary [file 41467_2024_49014_MOESM2_ESM.pdf]

nature portfolio

Corresponding author(s): Daniel Palanker, Leopold Schmetterer, Tong Ling

Last updated by author(s): May 12, 2024

Reporting Summary

Nature Portfolio wishes to improve the reproducibility of the work that we publish. This form provides structure for consistency and transparency in reporting. For further information on Nature Portfolio policies, see our Editorial Policies and the Editorial Policy Checklist.

Statistics

For all statistical analyses, confirm that the following items are present in the figure legend, table legend, main text, or Methods section.

n/a	Confirmed	
[ ]	[x]	The exact sample size (*n*) for each experimental group/condition, given as a discrete number and unit of measurement
[ ]	[x]	A statement on whether measurements were taken from distinct samples or whether the same sample was measured repeatedly
[ ]	[x]	The statistical test(s) used AND whether they are one- or two-sided<br>*Only common tests should be described solely by name; describe more complex techniques in the Methods section.*
[x]	[ ]	A description of all covariates tested
[x]	[ ]	A description of any assumptions or corrections, such as tests of normality and adjustment for multiple comparisons
[ ]	[x]	A full description of the statistical parameters including central tendency (e.g. means) or other basic estimates (e.g. regression coefficient) AND variation (e.g. standard deviation) or associated estimates of uncertainty (e.g. confidence intervals)
[ ]	[x]	For null hypothesis testing, the test statistic (e.g. *F*, *t*, *r*) with confidence intervals, effect sizes, degrees of freedom and *P* value noted<br>*Give P values as exact values whenever suitable.*
[x]	[ ]	For Bayesian analysis, information on the choice of priors and Markov chain Monte Carlo settings
[x]	[ ]	For hierarchical and complex designs, identification of the appropriate level for tests and full reporting of outcomes
[x]	[ ]	Estimates of effect sizes (e.g. Cohen's *d*, Pearson's *r*), indicating how they were calculated

Our web collection on statistics for biologists contains articles on many of the points above.

Software and code

Policy information about availability of computer code

Data collection	NI LabVIEW (19.0.1, National Instruments, TX, USA) was used to develop the image acquisition program.
Data analysis	MATLAB (2020a, 2021a, and 2022b, MathWorks, MA, USA) was used for OCT image processing, registration, and phase signal analysis. Python (3.9.12) was used for plotting figures. All the methods were described in detail in Methods and Supplementary Materials. The custom code for demonstrating our prolonged and multilayered optoretinography (ORG) method can be found at https://github.com/NTU-Ling-lab/ORG-Classification.

For manuscripts utilizing custom algorithms or software that are central to the research but not yet described in published literature, software must be made available to editors and reviewers. We strongly encourage code deposition in a community repository (e.g. GitHub). See the Nature Portfolio guidelines for submitting code & software for further information.

Data

Policy information about availability of data

All manuscripts must include a data availability statement. This statement should provide the following information, where applicable:

- Accession codes, unique identifiers, or web links for publicly available datasets
- A description of any restrictions on data availability
- For clinical datasets or third party data, please ensure that the statement adheres to our policy

All data needed to evaluate the conclusions in the paper are present in the paper and the Supplementary Information. Two example datasets, one corresponding to a 5-second recording with an acquisition rate of 200 B-scans/second and the other corresponding to a prolonged recording with an acquisition rate of 25 B-scans/

second, can be downloaded from https://github.com/NTU-Ling-lab/ORG-Classification. The raw experimental data are too large to be publicly shared, yet they are available for research purposes from corresponding authors upon request. Requests will be fulfilled within 2 months. Source data are provided in this paper.

Research involving human participants, their data, or biological material

Policy information about studies with human participants or human data. See also policy information about sex, gender (identity/presentation), and sexual orientation and race, ethnicity and racism.

Reporting on sex and gender	This study did not involve human participants, their data, or biological material.
Reporting on race, ethnicity, or other socially relevant groupings	This study did not involve human participants, their data, or biological material.
Population characteristics	This study did not involve human participants, their data, or biological material.
Recruitment	This study did not involve human participants, their data, or biological material.
Ethics oversight	This study did not involve human participants, their data, or biological material.

Note that full information on the approval of the study protocol must also be provided in the manuscript.

Field-specific reporting

Please select the one below that is the best fit for your research. If you are not sure, read the appropriate sections before making your selection.

- [x] Life sciences
- [ ] Behavioural & social sciences
- [ ] Ecological, evolutionary & environmental sciences

For a reference copy of the document with all sections, see nature.com/documents/nr-reporting-summary-flat.pdf

Life sciences study design

All studies must disclose on these points even when the disclosure is negative.

Sample size	No sample size calculation was performed in this study, as it is a pilot study without previous reports. The sample size was determined to be adequate for generating consistent data.
Data exclusions	Data with excessive motion artifacts (mainly due to heavier breathing effects of certain animals during anesthesia) were excluded from further analysis, because those excessive motion artifacts introduced out-of-plane movement, which our image registration algorithm could not correct.
Replication	The signal reproducibility test was performed as a separate experiment as a pilot study, and similar results were obtained with the same experiment conditions. In the reported results, multiple tests were conducted on different rats (n at least 3) for each experimental protocol, and similar results were obtained.
Randomization	Rats were randomly assigned to different study groups.
Blinding	Blinding was not considered in this study and was not applicable because this study did not compare different animal groups.

Reporting for specific materials, systems and methods

We require information from authors about some types of materials, experimental systems and methods used in many studies. Here, indicate whether each material, system or method listed is relevant to your study. If you are not sure if a list item applies to your research, read the appropriate section before selecting a response.

Materials & experimental systems

n/a	Involved in the study	
x		Antibodies
x		Eukaryotic cell lines
x		Palaeontology and archaeology
	x	Animals and other organisms
x		Clinical data
x		Dual use research of concern
x		Plants

Methods

n/a	Involved in the study	
x		ChIP-seq
x		Flow cytometry
x		MRI-based neuroimaging

Animals and other research organisms

Policy information about studies involving animals; ARRIVE guidelines recommended for reporting animal research, and Sex and Gender in Research

Laboratory animals	A total of 48 wild-type, Brown Norway rats (Rat Resources and Research Centre), aged between 6-16 weeks, were used for this study, and a detailed description was included in the Supplementary Table S2.<br>Five (Male = 3) rats, aged 12 weeks, were used to pre-train the classifier (Fig. 2 and Fig. 3a)<br>Twelve (Male = 10) rats, aged between 6 and 16 weeks, were used for dark adaptation experiments (Fig. 3b).<br>Fifteen (Male = 10) rats, aged between 6 and 12 weeks, were used for light adaptation experiments (Fig. 3c).<br>Nine (Male = 6) rats, aged between 6 and 12 weeks, were used for prolonged recording (Fig. 1 and Fig. 6).<br>Seven rats (Male = 4), aged between 6 and 10, were used for volumetric recording to generate en-face visualization (Fig. 5).
Wild animals	The study did not involve wild animals.
Reporting on sex	Both male and female animals were included in this study.
Field-collected samples	This study did not involve samples collected from the field.
Ethics oversight	All experiments were conducted in accordance with guidelines and approvals from the Institutional Animal Care and Use Committee (IACUC), SingHealth (2020/SHS/1574).

Note that full information on the approval of the study protocol must also be provided in the manuscript.

Plants

Seed stocks	This study did not involve any plant.
Novel plant genotypes	This study did not involve any plant.
Authentication	This study did not involve any plant.
