# Supplementary material for: Light-evoked deformations in rod photoreceptors, pigment epithelium and subretinal space revealed by prolonged and multilayered optoretinography
Source: Nat Commun. 2024 Jun 19;15:5156. doi: 10.1038/s41467-024-49014-5 (PMC11186825; doi:10.1038/s41467-024-49014-5)
Supplement: Supplementary file 3 — Description of Additional Supplementary Files [file 41467_2024_49014_MOESM3_ESM.pdf]

Supplementary Movie 1:

En-face temporal evolution of the outer segment (OS) and subretinal space (SRS) signals. Scale bar: 100 μm.
